# Reasons for informal payments from the perspective of health care providers and recipients: a qualitative study in Iran

**DOI:** 10.1186/s41256-022-00263-1

**Published:** 2022-09-01

**Authors:** Mohammad Arab, Bahman Khosravi, Hossein Safari, Hojat Rahmani, Ghasem Rajabi Vasokolaei, Mohammadreza Mobinizadeh, Farhad Habibi

**Affiliations:** 1 Department of Health Management and Economics, School of Public Health, Tehran University of Medical Sciences, Tehran, Iran; 2 Health Promotion Research Center, Iran University of Medical Sciences, Tehran, Iran; 3 National Institutes for Health Research, Tehran University of Medical Sciences, Tehran, Iran

**Keywords:** Informal payments, Health systems, Health care providers, Health care recipients

## Abstract

**Background:**

Informal payments are one of the major obstacles to health system reform in many developing countries, and its elimination is on the agenda of health system policymakers in many countries, including Iran. This study was conducted to identify the causes of informal payments in the Iranian health system.

**Methods:**

This was a qualitative and exploratory study. The study environment included the Ministry of Health, physicians' offices, medical universities, and hospitals and health centers. The study population included health care providers (physicians and hospital staff, managers, supervisors, and nurses) and health care recipients (patients or patients who had a history of dealing with informal payments). Data were collected using open-ended questions and semi-structured interviews. Snowball sampling method was used to select managers, chief executive officers (CEOs) and nurses. Convenience sampling was used to select physicians due to their lack of participation and cooperation. Content analysis method was used to analyze the data.

**Results:**

Reasons for informal payments were divided into 4 themes including: Economic factors (improper tariff valuation of services; failure to increase tariffs proportionate to inflation; lack of comprehensive participation of stakeholders in determining tariffs; tariff inconsistency in the public, private and charity sectors; etc.); socio-cultural factors (decreased social capital of the medical community among the people; improving the quality of life; incorrect comparison of providers' income levels with the income of doctors in other countries; existence of a culture of gratitude and appreciation; health as a priority for society; pride of service recipients; pride of service providers; etc.); service delivery challenges (high professional skills of the doctor; use of modern medical equipment; the monopoly of some doctors, etc.) and legal-political factors (inadequate monitoring by upstream organizations; lack of strict rules; difficulty of proving informal payments; presence of stakeholders in management and policy making processes)**.**

**Conclusions:**

Knowing the causes of informal payments can help reduce or eliminate it. The results of this study identified the causes of informal payments in the Iranian health system. Accurate knowledge of the needs and motivations of both health care providers and recipients can be effective in accurately identifying and eliminating this phenomenon.

## Introduction

Informal or under the table payments in the healthcare sector are costs that are received from the patient outside the framework of formal tariffs and are a very common phenomenon in the Iranian healthcare system [1, 2]. Undoubtedly, any undefined financial relationship between healthcare providers and patient will not only cause a serious problem to the physician–patient relationship, but over time will cause a serious problem to the trust between the community and the medical team [3]. Some studies have shown that the quality of clinical services is also affected by informal payments [4, 5]. In addition to its impact on individuals, informal payments will also affect the performance of the healthcare system. Its effect on the health care system will be revealed through its impact on the distribution of services and the allocation of resources in this sector. These types of payments can also have positive effects on the health system. Reports, for instance, show that even a small amount of money can motivate physicians to continue working in the public sector [6, 7]. When health system financing is largely financed by informal payments, providers find sufficient incentive to provide more attractive but unnecessary services that lead to greater production inefficiencies [8].

Informal payments act as a deterrent on the way of access to and use of health services and system efficiency, as well as an obstacle to health reform [9]. These payments not only increase out-of-pocket payments, they may also escalate the catastrophic healthcare expenditures and poverty among the poor, and can have negative effects on justice and health, and in the case of those people with insurance who should pay such payments, it raises doubts about financial protection issues [10]. These payments are one of the major obstacles to health system reform in many developing countries, and its elimination is on the agenda of health system policymakers in many countries, including Iran. Financing in the Iranian health system is a combination of public resources (government budget), social health insurance, private (supplementary) insurance, and out-of-pocket payments of citizens [11]. In Iran, OOP payments account for more than 50% of the total health care costs of the country [12]. Out-of-pocket payment is the most descending method of paying for health and is the method that leads to the most exposure to catastrophic financial risks for individuals [13]. Informal health payments are part of out-of-pocket payments that are made directly by patients and their families. These payments have become a public policy concern for low- and middle-income countries and even for developed countries in Europe and Asia because of their negative consequences [14]. Studies conducted in Iran also show the high prevalence of such payments for health services [1, 15, 16]. Therefore, considering the importance of the issue and in order to help health system planners and policy makers plan and make reforms in this field, this study was conducted to identify the causes of informal payments in the Iranian health system.

## Methods

### Study design

This qualitative and exploratory study was conducted to identify the causes of receiving and paying under the table payments from the perspective of providers and recipients of health services. In this study, informal payments are amounts of money or anything related to gifts, etc. that are taken outside the defined system of tariffs in the health system or the book of relative value of health services by those providing medical and paramedical services from people in need of health services. The study population included the Ministry of Health, physicians' offices, medical universities, and hospitals and health centers.

### Participants

The study population included health care providers (physicians and hospital staff, managers, supervisors, and nurses) and health care recipients (patients or patients who had a history of dealing with informal payments). The criteria for selecting the providers were having more than 5 years of work experience, knowledge of the subject and interest in participating in the interview. Also, in the selection of physicians, an attempt was made to include those in the study who had a history of receiving informal payments, for which the opinion of experts as well as policymakers participating in the study was used. The criteria for selecting recipients were having an informal payment or dealing with such payments. Having a bachelor's degree or higher and an interest in participating in the interview were other criteria for selecting health care recipients. Also, based on the information of 1690 system (system related to patients' complaints about informal payments), an attempt was made to use the participation of patients who referred to specialists with the highest rate of complaints such as orthopedists, obstetricians, neurologists and cardiologists.

### Data collection and processing

Data were collected using open-ended questions and semi-structured interviews. In order for the questions of the interview to be acceptable, consistent, verifiable and transferable, the opinions of experts were first asked in this regard, and after determining the questions, three people from each group were selected and after the interview, the questions were reviewed again. Convenience and snowball sampling methods were used to select the providers. Snowball sampling method was used to select managers, Chief executives officers (CEOs) and nurses. Convenience sampling was used to select physicians due to their lack of participation and cooperation. However, in some cases, physicians were selected through being introduced by their colleagues. Finally, data were saturated after 16 interviews. Convenience sampling was used to select recipients with a history of informal payments. Interviews were conducted with 12 people who had a history of paying informal payments.

### Data analysis

Content analysis method was used to analyze the data. Collection, analysis and transcription of the data were performed simultaneously. After the interview, all the interviews were implemented and a list of themes and sub-themes were prepared. In the next step, the framework of specific themes was categorized and indexed. All these steps were performed using MAXQDA-10 software.

## Results

### Descriptive statistics

In the present study, 6 participants were physicians, 4 were hospital managers or CEOs, 4 were treatment or technical managers, 2 were nurses and 12 were patients or their companions (Table 1).

**Table 1:** Demographic characteristics of the study participants

Study population	Interviewee	Nos.
Service providers	Doctor	6
	Hospital CEO/manager	4
	Treatment manager/technical assistant	4
	Nurse	2
Service recipients	Patient	12

Table 2 shows the amount of informal payments and the type of medical specialty to which the payment was made. A total of 40,200,000 Tomans was paid informally to various specialties during 1 year. Among the various specialties, obstetricians and gynecologists received the highest amount of informal payments.

**Table 2:** Data related to the amount of payment and type of specialty

Name of the person	Sex		Amount of money (IRR)	Specialty of the doctor
	Male	Female		
First person	*		IRR 6,000,000	Obstetrics
Second person		*	IRR 70,000,000	Orthopedics
Third person		*	IRR 180,000,000	Cardiology
Fourth person		*	IRR 40,000,000	Oncology
Fifth person		*	IRR 25,000,000	Oncology
Sixth person	*		IRR 3,000,000	Obstetrics
Seventh person	*		IRR 20,000,000	Orthopedic spine surgeon
Eighth person	*		IRR 20,000,000	Neurology
Ninth person	*		IRR 20,000,000	Maxillofacial surgery
Tenth person	*		IRR 5,000,000	Obstetrics
Eleventh person	*		IRR 8,000,000	General surgery
Twelfth person	*		IRR 5,000,000	Obstetrics
Total	8	4	IRR 402,000,000	

### Qualitative findings

The findings in Table 3 show the reasons for informal payments from the perspective of health care providers. In this section, the interviewees were asked questions about the reasons for receiving under-the-table payments. After analyzing the data, a total of 4 main themes and 23 codes were obtained.

**Table 3:** Reasons for informal payments from the perspective of health care providers and recipients in Iran

Main themes	Codes
MT1: economic factors	Improper tariff valuation of services Failure to increase tariffs proportionate to inflation Lack of comprehensive participation of stakeholders in determining tariffs Tariff inconsistency in the public, private and charity sectors Failure to pay financial claims on time by insurance companies Insufficient coverage of basic and specialized services by insurance companies
MT2: socio-cultural factors	Decreased social capital of the medical community among the people Improving the quality of life Incorrect comparison of providers' income levels with the income of doctors in other countries Existence of a culture of gratitude and appreciation Health as a priority for society Pride of service recipients (luxury in receiving services) Pride of service providers (merit in providing services) Lack of attention to ethical-religious issues by both providers and recipients of services Insufficient attention to teaching professional ethics at universities and the workplace
MT3: service delivery challenges	High professional skills of the doctor Use of modern medical equipment The difficulty of the medical profession and the lack of attention to the needs of the medical community during education The monopoly of some doctors
MT4: legal-political factors	Inadequate monitoring by upstream organizations Lack of strict rules Difficulty of proving informal payments Presence of stakeholders in management and policy making processes

#### MT1: economic factors

Economic factors were considered as one of the main causes of the prevalence of informal payments from the perspective of health service providers in the Iranian health system. After analyzing the data, this theme and its causes were categorized into 6 main causes as follows.

##### Improper valuation of service tariffs

Improper or unrealistic valuation or tariffing of services, both in the private and public sectors, from the point of view of health care providers, and especially the physicians participating in the study, results in higher informal payments to their colleagues. The providers were of the opinion that the tariffs had been increased with the implementation of the health system transformation plan, but it was still far from the actual amount.

“Medical expenses, i.e. low medical salaries, are one of the reasons why it may have existed from the beginning, and doctors consider their own salary higher than what is given to them in the government system. Therefore, they want to compensate this low income and the easiest way is to get under the table payments. (D-3)”

##### No increase in medical tariffs proportionate to inflation

As pointed out by health service providers, economic instability and high inflation in the country, not only in the health sector but also in other organizations and sectors, cause illegal and unconventional earnings due to compensation for lost income caused by inflation. One of the participants stated:

“One of the reasons for informal payments and in fact the reason for having it was the fixed medical and surgical tariffs despite the inflation rate. The medical salary, which is, for example, 38 thousand Tomans should be increased to 50 or 60 thousand Tomans. (A-7)”

##### Lack of comprehensive participation of stakeholders in setting tariffs

Weak involvement of stakeholders in the tariff process and excessive government interference in this process leads to unrealistic tariffs and this ultimately results in an increase in paying informal payments by patients. Such issue existed before the health transformation plan and still exists after the transformation plan and has become even worse.

“Government interference in tariff process, however, in most jobs that are not even related to doctors, is that, for example, you go to a mechanic, a pizza shop or a locksmith, they put a price tag there. Who sets this price tag?" "The guild and the union have done this. Almost all the guilds set the tariff themselves in proportion to what they do, while here the doctors themselves have little role in determining the price. (1–8)

##### Not having the same tariffs in public, private and charity sectors

Participants cited unequal tariffs in public and private sectors, which lead to injustice, as one of the reasons for receiving informal payments. One of the participants said in this regard:

“I think another reason for receiving informal payments is the difference between the private and public sectors in terms of medical tariffs. As a result, the doctor who works in the public sector compares the tariff with the private sector and sees that this tariff is much lower than the private sector. This makes him either takes money from the patient for what he wants to do there, or direct the patient to the private sector, which has both higher tariffs and less supervision, and it is easier for him to take the money he wants from the patient. (A-11)”

##### Failure to pay financial claims on time by insurance companies

One of the main reasons for informal payments from the perspective of physicians and after the implementation of the health system transformation plan is the lack of timely payment by insurance organizations. Although this delay existed before the implementation of the transformation plan, from the people's point of view, this delay could not cause the expansion of informal payments in the health system.

“When a surgeon goes to the hospital in the middle of the night and operates on a patient, but he/she is paid a small fee a few months later, this will tempt the doctor to ask for informal payments. (A-2)”

##### Insufficient coverage of basic and specialized service packages by insurance companies

According to health service providers, lack of trust in insurance organizations, which is due to them not paying hospital claims on time and also providing inappropriate service packages, has led to the spread and expansion of informal payments before and after the health system transformation plan in Iran. With the implementation of the health system transformation plan, this cause has become more prominent than before. One of the participants said in this regard:

“If the insurance cards were valid and accepted in public hospitals, the patient would no longer be willing to give informal payments. In this case, the surgeons who receive informal payments should also look for the patient. (1-7)”

#### MT2: socio-cultural factors

After interviewing the participants, socio-cultural factors were divided into 9 categories.

##### Decreased social capital of medical community among people

Some providers believed that due to excessive complaints from physicians, which cause physicians to spend their time resolving such issues, physicians are receiving informal payments to compensate for the costs incurred.

“People can complain about doctors in any way. Failure to do proper medical practice, even if the doctor is not to blame, is a way for people to complain and this is bad, because, with the current situation, every doctor is now trying to prevent from a problem. The doctor also tries to have a financial base that if someone complains, at least he can be held accountable. (A-4)”

Some providers also believed that the negative atmosphere created by negative media advertisements and programs would cause their colleagues to receive informal payments. Doctors said that when there is this unfair negative publicity and atmosphere and people do not have a good view of doctors, doctors unconsciously get affected by this atmosphere and behave in an unusual way.

“It may not be as widespread as the advertisements in the media. When there is a negative atmosphere against the doctors and people no longer trust and accuse us, well, my colleagues say, now that whether we receive informal payments or not, this atmosphere against us won't change, so it's better to receive it, and this is why they get informal payments from the patients and this atmosphere gets worse every day. (A-5)”

##### Improving the quality of life

Some providers receive informal payments because of their higher quality of life, which is due to cultural problems in the community. Having a higher quality of life has always existed in other professions and organizations, but the reason and method of achieving it has been different. According to the providers, one of the reasons for having a higher quality of life is the society's excessive expectation of doctors' standard of living.

“In fact, we graduated after 12 or 13 years of education, and if we drive a simple car after this period, people will tell us that someone is driving a simple car after all these years of education, and this subconscious pressure will lead doctors to receive informal payments. (1-6)”

##### Incorrect comparison of providers' income levels with their counterparts in other countries

One of the issues raised by physicians that have led to receiving informal payments is the unintentional comparison of their income with that of physicians in other countries.

“Besides that is the comparison of Iranian doctors to doctors in other countries. They always compare themselves with other countries and when see the financial differences; they try to make it up through other ways. (A-15)”

##### Existence of a culture of gratitude and appreciation

The culture of gratitude has always existed and will exist in the culture of our country, but its method will be different in different people. Some physicians believed that patients themselves made additional payments voluntarily in order to compensate for the physicians' efforts.

“I was talking to one of my colleagues who was saying, 'Why shouldn't I get the money. The patient came to me, I said that I want to operate on your knee, he said how much is it, Mr. Doctor? I said, for example, 4 million Tomans, he said you want to do it yourself or your student will do it for me. Then he said 4 million is very little. I will give you 10 million and if you do the operation well, I will give you 5 million more after the operation. (A-2)”

##### Health is a priority for society

Some providers argued that because some people may only get sick once in a lifetime, it is best to regain their health as best as they can, and therefore make illegal payments to achieve their goals at the request of a physician or of their own free will.

“I myself did not resist when I saw that the doctor wanted informal payments. How many times do I want to have children? I pay more but I am sure about the health of my wife and child. I was saying to myself what if I don’t pay this money and something happens to my wife and child. (D-6)”

##### Pride of service recipients (luxury in receiving service)

The existence of a culture of luxury in some recipients of services regardless of receiving quality services was another reason for the existence of informal payments in the Iranian health system. A culture called "competition" culture, which makes people in Iran refer to the famous doctors of the city, who are even famous for receiving informal payments, in order to show off their pride and power, and even voluntarily pay more money outside the defined framework.

“Unfortunately, there is this culture of competition in our country. We had a relative whose wife wanted to give birth to a child in the famous hospital of the city and by a very famous doctor. He himself knew that the doctor would take under the table payments too much, but because his wife had said that I must go to that doctor, he had to take her there and had to pay a lot of money as well. (D-7)”

##### Pride of service providers (merit in providing services)

There is a culture of merit among health system providers in order to increase the value of services and show the quality of services provided by them, which leads to receiving informal payments from the clients.

“Another problem is that many of our doctors take bribes to attract customers. You may say what do I mean? I will explain. You see, some of our doctors, many of whom are professors, open their offices in luxurious and expensive places in the city and then try to say that our work is very good and that we are skilled so you have to stay in waiting list and if you want to operate, you have to go to a private hospital. There, you have to pay some money to the hospital and also I get a certain amount of money for the operation (P-11).”

##### Lack of attention to ethical-religious issues by both providers and recipients of services

Considering the religious conditions of Iran and the fact that many religious authorities have forbidden receiving informal payments by doctors, this issue was reflected in the findings of the present study. According to some providers, due to the weak religious beliefs that exist in some providers and lack of adherence to religious principles, they easily receive informal payments. One of the providers stated:

“In my opinion, if a doctor has a basic belief, he will not do it. If a doctor has a religious basis, from a religious point of view, he/she does not do this, but if they do not have a belief, they do so easily. Some of the worst kinds, in my opinion, are those who accept morality and religious issues but justify themselves and justification is something that I, as a physician, may inadvertently have the same justification for the next ten years and think that I am doing my work and it is not wrong, but when I go back and look from the outside, I see that it is informal payment”. (A-5).

##### Insufficient attention to teaching professional ethics at universities and the workplace

According to the providers, lack of training or poor ethics training in medical courses at university, and choosing the wrong role models by physicians are one of the main reasons for receiving informal payments.

“So far, I have not heard anything from any professor about whether to take informal payments or not, but we have heard this many times that the tariffs and fees of a doctor are low, and most of us look at the performance of those professors, professors or anyone else have a different perspective. Someone takes a teacher as a role model and treats like them. (A-6)”

#### MT3: service delivery challenges

This theme includes four reasons for having informal payments as well as items related to the methods of treatment and unbalanced distribution of physicians in different places.

##### High professional skills of the doctor

The providers believed that the skills of physicians in their work would cause them to receive informal payments.

“For example, a doctor who is very knowledgeable in heart surgery says, "Why should I get the same amount of money like a usual heart surgeon who is not skilled in his/her work one tenth of me?" I worked so hard and have done many surgeries to get here, but I still have to get the same amount of money like a typical surgeon who has just started and it is not clear what he/she will do. (A-13)”

##### Use of modern medical equipment

Using various treatment methods was mentioned as one of the reasons for having informal payments in the Iranian health system. The providers believed that the facilities and equipment that some physicians use in private offices or hospitals were up-to-date and available to a small number of people, and therefore considered the current approved prices to be unfair.

“In big cities, the costs are generally higher and their techniques and methods are also different, and these have an impact on receiving informal payments. When a doctor is using a medical device which is very expensive, he/she does the math and understands that his/her income doesn’t match the costs; therefore, they get more money from the service recipients. Well, these devices and equipment are usually used in big cities and places where people are rich, and the people there have no problem with the costs, so they give whatever the doctor wants. (D-8)”

##### The difficulty of the medical profession and the lack of attention to the needs of the medical community during their education

From the physicians' point of view, the difficulty and the length of studying medical courses are among the reasons for having informal payments in the Iranian health system.

“Well, some colleagues believe that we studied for so many years, stayed up so many nights, did not receive any money before graduation, and now we are 40 years old, and nothing can be done with these tariffs. After all this study, we have to go through hard night shifts again, stay on foot in the operating room for a few hours, and if there is a problem for the patient, we have to answer to everyone. When they look at this, they feel that they deserve more than this. (1-4)”

##### The monopoly of some doctors

The monopoly of some physicians, especially in deprived areas, and giving patients the right to choose is one of the main reasons for receiving informal payments before and after the health system transformation plan in Iran.

“In our city, the highest informal payments are received by the medical group, who are 2 doctors in the whole city, so the supply limit is very important. But in a field where there are 10 or 15 specialists in the city, the proportion of informal payments decreases to the same extent .(1-9)”

#### MT4: legal—political factors

Participants in this part of the study pointed out four legal and political reasons to be effective in promoting and prevailing informal payments in the Iranian health system.

##### Inadequate monitoring of upstream organizations

Most of the participants in the study believed that poor monitoring, both in the past and now, was one of the main reasons for the formation and prevalence of informal payments in the Iranian health system.

“The issue of monitoring is very important and effective here, and I think that one of our serious problems is the gaps in monitoring of and dealing legally with violators. (A-10)”

##### Lack of strict rules

The findings of the present study showed that the lack of strict and deterrent laws before and after the implementation of the health system transformation plan has caused having informal payments in the Iranian health system. Most people believed that the laws became stricter after the implementation of the health system transformation plan, but it still does not have enough deterrence to eliminate the phenomenon of informal payments in Iran.

“My many years of experience in this field show that we have no effective mechanism to deal with the phenomenon of under the table. In your opinion, now if one of my colleagues takes informal payments and it is proven, what will happen.(1-8)”

##### The difficulty of proving the receiving of informal payments

Another reason for the prevalence of informal payments in the Iranian health system is related to the difficulty of proving it, which remains in force even after the implementation of the health system transformation plan.

“Proving that the provider asked the service recipient for informal payments is a difficult issue for the person who gave it; for example, you have to prove that you gave informal payments and the provider denies it. (A-9)”

##### Presence of stakeholders in management and policy-making processes

The presence of stakeholders and providers at the levels of management and policy making in the health system, as well as the political power and skills of some of them leads to receiving informal payments by them. One of the participants said:

“In my opinion, the health transformation plan has not been able to prevent this very much. For example, there are doctors who have a power of their own that they are not afraid of the consequences. I think such things happen behind the scenes. Iran Medical Council are all physicians and all of them support physicians, and the rest of them, who are either connected somewhere or do their job well enough that nobody can say anything to them, they get informal payments without any fear, and everyone knows that. (A-16)”

## Discussion

The aim of this study was to identify the causes of informal payments from the perspective of health care providers and recipients. One of the reasons for the informal payments was economic challenges. Improper tariff valuation of health services was one of the main reasons for the prevalence of informal payments [15, 17]. In studies conducted in Iran [18] Turkey [19], Tanzania [4, 20], Hungary [21], and Central and Eastern European Countries [22], health care providers cited low incomes and wages as the main reasons for receiving informal payments. Another reason given by doctors was the existence of injustice in tariffs of various medical groups. In a study conducted in Albania [23], the injustice among health personnel payments was mentioned as one of the causes of the prevalence of informal payments in this country.

Inadequate coverage of services by insurers was one of the reasons for receiving informal payments in Iran. In studies conducted in Iran [24] and Tanzania [4], poor performance and incomplete insurance coverage were cited as reasons for receiving under the table. On the other hand, a study conducted in China showed that social health insurance coverage significantly increases the likelihood of paying informal payments by hospitalized patients, while private health insurance coverage significantly reduces this likelihood [25]. It seems that determining the package of appropriate health services in cooperation with the ministry of health and the ministry of cooperatives, labor and social welfare can be of great help in this regard.

Socio-cultural challenges were among the reasons for having informal payments. One of the social and cultural reasons for receiving informal payments from the perspective of providers was to improve the quality of life. Providers, especially physicians, stated that their income was lower than that of physicians in other countries. A study conducted in Moldova [26] showed that in order to have a living higher than the standard, physicians receive informal payments. This finding is consistent with the findings of the present study in terms of achieving the expected income and having a higher quality of life. It seems that when a doctor has an insatiable craving for income, that doctor does not do scientific and educational work, and according to world standards, the activity of these doctors should be prevented.

Cultural customs and beliefs of the society such as the culture of gratitude and appreciation are another reason for making informal payments. Culture and customs of the society and the existence of the culture of gratitude and appreciation were mentioned as the reasons for patients paying under the table in Turkey [19], Tanzania [4, 9], Hungary [27], Moldova [26] and Bulgaria [28]. In a study conducted in Iran, some patients stated that they made such payments voluntarily in order to appreciate the efforts of medical staff [29]. A study conducted in Greece also showed that appreciation, monopoly, and reputation were among the causes of informal payments [30].

One of the socio-cultural challenges was the boasting of doctors and patients, which can be said to be one of the rare reasons for receiving and paying under the table. Some physicians feel that if they do not request such payments, they will lose their patients because patients feel that their physician will not provide them with quality services. This issue is completely rooted in the culture of the Iranian people and it seems that the plan to transform the health system cannot be effective in this regard and the need for culture building is seen at the macro level of the country [31].

Lack of attention to ethical-religious issues by both service providers and recipients is another reason for having informal payments. From religious point of view, it is illegitimate to take informal payments. Although some jurists have considered it permissible to receive this payment if it is accompanied by the patient's consent, it seems to be invalid because the patient's consent is accompanied by the abuse of the emergency situation in which the patient is [32].

According to the findings of the present study, service delivery challenges were another cause of informal payments. Reputation and skills, which themselves may be due to the use of new treatment methods and equipment, were also cited as factors influencing the prevalence of informal payments. Health care providers believed that there was an injustice in tariffs and payments for highly skilled physicians and physicians who use up-to-date and expensive treatment methods and equipment, and this injustice has manifested itself in the form receiving illegal payments. It seems that with the cooperation of the Ministry of Health and insurance organizations and even through the country's customs, doctors who try to import and use up-to-date medical equipment can be prevented from trying to receive extra money illegally by giving financial discounts.

In the case of physicians with ability and work experience, it may be possible to use the rating or accreditation system of Iranian hospitals and classify physicians into different classes based on quantitative and qualitative performance factors and use different tariffs for groups with different skill levels in each specialty. In studies conducted in Turkey [19] and Hungary [21], patients paid informal payments in order to receive complex medical services and treatment by renowned physicians. According to the findings of the present study, one of the reasons for the prevalence of informal payments is the monopoly of some physicians or medical specialties. In studies conducted in south Asia, the exclusive power of providers was cited as one of the main reasons for the prevalence of informal payments [33]. With the implementation of the health system transformation plan and the allocation of physicians to deprived areas, this problem has largely been resolved in public sector, but in private sector, there is still a need for special attention by policymakers.

In the present study, legal and political weaknesses became one of the main reasons for the prevalence of informal payments in the Iranian health system. The most important legal and political weaknesses are the inadequate monitoring of upstream organizations, the difficulty of filing a complaint, the lack of strict rules, the difficulty of proving informal payments by patients, and the presence of stakeholders in management and policy-making levels. In various studies, the lack of decisive supervision on activities of physicians by regulatory institutions, lack of determination in handling people's complaints for various reasons such as excessive compliments and negligence in dealing with physicians due to the reason that health executives and physicians are both doctors, and lack of attention of officials to the issue and inability to address the issues [2, 31].

It seems that increasing supervision and coordination between the medical council, ministry of health ‌, and ministry of cooperatives, labor and social welfare will be effective in solving the problem of receiving informal payments from patients. In studies conducted in Hungary [21] and Turkey (19), the weakness of the monitoring system was cited as a factor influencing the prevalence of informal payments, which is similar to the causes found in the present study.

This study has some limitations as follows:

- Some patients were conservative in answering questions or were reluctant to record their actual opinions. We tried to solve the problem by justifying them about the purpose of the study and making sure that the opinions of all of them were considered confidential.
- Lack of cooperation of relevant organizations in providing information. The problem was partially resolved with the help and guidance of supervisors and consultants.
- Doctors' lack of cooperation due to the sensitivity of the issue, which was somewhat resolved by their justification that all information was confidential.
- Doctors dodge answering questions to support colleagues and the medical community.
- Lack of cooperation of some policy makers and doctors due to being busy, which in some cases was solved with the help of supervisors and consultants.

## Conclusions

Knowing the causes of this phenomenon can help reduce or eliminate it. The results of this study identified the causes of informal payments in the Iranian health system. Accurate knowledge of the needs and motivations of both health care providers and recipients can be effective in accurately identifying and eliminating this phenomenon. A careful understanding of the effects of these payments by policymakers can lead to more appropriate decisions in this area as a policy option that is agreed upon by all. It can be acknowledged that increasing the salary of providers based on their performance and skills, and in fact the implementation of performance-based payment in the country's health system, will help policymakers reduce this phenomenon in Iran. Professional ethics training is a way to gain trust and understanding between people as well as a means to show the ugliness of bribery.

## Data Availability

The datasets used during this study are available from the corresponding author on reasonable request.

## Abbreviation

CEOs: Chief executives officers
